# HOW DO HEALTH CARE PROVIDERS VIEW HOSPICE CARE IN BINZHOU, CHINA?: A QUALITATIVE INQUIRY

**DOI:** 10.1093/geroni/igae098.2389

**Published:** 2024-12-31

**Authors:** Heshuo Yu, Kate de Medeiros

**Affiliations:** Fudan University, Shanghai, Shanghai, China (People’s Republic); Concordia University, Montreal, Quebec, Canada

## Abstract

Although many large Chinese cities have begun hospice services, it is still a relatively new concept in many parts of the country, especially in smaller cities. The purpose of this study was to gain a better understanding of health care providers’ (physicians and nurses) attitudes toward hospice care, to include perceptions of barriers and facilitators of implementation in a fourth-tier city. Using a qualitative descriptive approach, semi-structured, open-ended interviews were conducted with 15 health care providers. Two major themes were identified: 1) barriers to developing hospice care and 2) prospective facilitators to develop hospice care. There is currently much ambiguity regarding what governmental agency would oversee hospice care if implemented, who would be responsible for payment, the importance of developing interdisciplinary care teams, and concerns about worker shortages. Future research is encouraged to investigate hospice attitudes across different local healthcare systems and to encourage the development of local hospice support.

